# Optimization of icterus interference thresholds for 45 parameters on the Atellica analyzers CH930 and IM1300

**DOI:** 10.11613/BM.2026.020707

**Published:** 2026-06-15

**Authors:** Damien Leleu, Damien Denimal, Laurence Duvillard

**Affiliations:** Université Bourgogne Europe, CHU Dijon Bourgogne, Service de Biochimie, INSERM UMR 1231, Dijon, France

**Keywords:** Atellica analyzers, icterus interference, icterus index

## Abstract

**Introduction:**

The data provided by the manufacturer regarding icterus interference on the various parameters measured on Atellica analyzers are very incomplete, potentially leading to erroneous result interpretation. We aimed to evaluate icterus interference on Atellica CH930 and IM1300 for 45 parameters and to suggest revised icterus indexes for a better interpretation of results.

**Materials and methods:**

Pools of serum or plasma were spiked with various concentrations of either conjugated or unconjugated bilirubin. Analytes were measured in triplicate on bilirubin-spiked and native samples. Biases were calculated as the difference between the mean results of both samples. We compared the observed biases to the threshold of 10% similar to the manufacturer, and also to the total allowable errors recommended by the European Biological Variation Study (EuBIVAS).

**Results:**

Based on significant bias above 10%, revised icterus index thresholds were higher than manufacturer recommendations for 33 parameters, and lower for 3 parameters. Based on the EuBIVAS criteria, the thresholds were higher than manufacturer recommendations for 33 parameters, and lower for 5 parameters. The revised thresholds differed between the 2 evaluation methods in 13 cases, being less strict for some parameters with the EuBIVAS criteria, and stricter for other ones. For some parameters, the magnitude of interference differed for conjugated and unconjugated bilirubin, or depended on the concentration of the analyte.

**Conclusions:**

This work provides important data that can be used to improve the interpretation of results for 45 parameters on Atellica analyzers CH930 and IM1300 in patients with hyperbilirubinemia.

## Graphical abstract

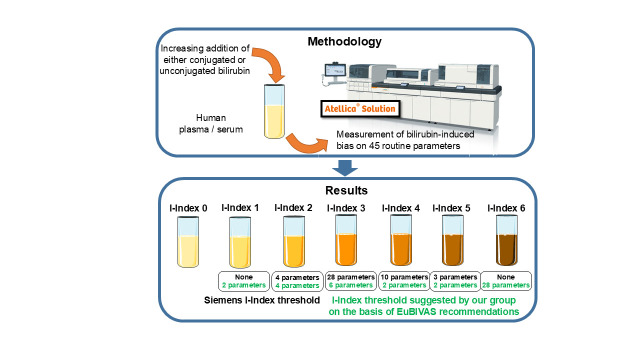

## Highlights

• Data from the supplier for icterus-induced interference on Atellica are very partial

• We evaluated it for 45 parameters by spiking samples with conjugated or unconjugated bilirubin

• The measured biases were compared to EFLM recommendations

• Icterus index threshold warrants to be revised for 38 among the 45 parameters tested

• Our data enable a more reliable interpretation of results for icteric samples on Atellica

## Introduction

Icterus is frequent for serum or plasma samples, especially in hospitalized patients (*1*). It is likely to interfere with the quantification of different biochemical parameters (*2*). Several mechanisms are likely to explain icterus interference in biochemistry assays (*2*). Firstly, since bilirubin absorbs light between 400 and 540 nm, it can interfere with colorimetric assays taking absorbance measurement at these wavelengths. Secondly, bilirubin can interfere with oxidoreduction reactions like Trinder reaction. Bilirubin is likely to reduce hydrogen peroxide in Trinder reaction, decreasing the reduction of the chromogen, resulting in a negative bias, which amount depends on the efficiency of hydrogen peroxide acceptor. But, for unclear reasons, the conjugated and unconjugated forms of bilirubin may induce interference in opposite directions.

It is highly important to understand the extent to which icterus interferes with the measurement of the concentration of an analyte. Indeed, laboratory professionals can either decide to cancel a result in case of significant icterus interference, or to transmit the result with either an indication of the direction or the approximate magnitude of the bias (*3*). Current analyzers are now able to evaluate icterus levels in samples through spectrophotometric measurement, with results reported as the icterus index (I-index). Unfortunately, some manufacturers have not always evaluated icterus interference across the full range of bilirubin concentrations encountered in patients (*4*). For the sake of caution, they recommend suppressing the results for samples whose I-index is above the threshold for which they stopped interference assays, even if there is no significant interference for that threshold. The problem described here above concerns Atellica analyzers (Siemens, Erlangen, Germany) for both chemistry and immunoassays (*5*). For some parameters, icterus interference is specific to one analytical platform and the I-index scale is heterogeneous among analytical platforms (*6*). As a result, data cannot be generalized from one platform to another.

The aim of the present study was to evaluate on the recently launched Atellica analyzers the icterus interference on the 45 parameters routinely quantified in our laboratory for which the manufacturer recommends to flag results from an icterus index between 1 and 5, suggesting a potential interference, whereas for most of these parameters, no significant interference was evidenced for the recommended thresholds (*i.e.* no interference higher than 10%, which is the criteria chosen by the manufacturer). We conducted interference experiments using both conjugated and unconjugated bilirubin since the two forms of bilirubin are likely to have varying effects on the results (*2*). For the parameters biased by icterus, we estimated the amount of interference for different I-index values to guide laboratory professionals in interpreting and potentially transmitting the result.

## Materials and methods

This analytical interference study was conducted between January 2024 and March 2025 in the Biochemistry department of the Dijon Bourgogne University Hospital.

### Materials

The effect of icterus was evaluated on 45 parameters using pools of serum, heparinized plasma, or plasma with sodium fluoride (from blood collected in BD Vacutainer SST™, PST™, and fluoride tubes with gel separator, respectively), which were kept at + 4 °C after routine analysis, for less than 36h. The nature of the biological matrix depended on the usual practices of our laboratory and was validated by the Atellica manufacturer as indicated in the technical sheets (*5*). Ethics committee approval and informed consent were not required in compliance with our institutional guidelines for the use of pools of anonymized leftover patient samples that were scheduled for disposal. Hemolysis, icterus and lipemia indices of plasma or serum pools were equal to 0 before spiking.

Interference was tested at concentrations whose choice is precised in Supplementary table 1. A specific pool (3 to 5 mL) was constituted just before experiments for each analyte and each concentration, from 3 to 5 samples. Interference was first tested for a low concentration of the analyte, and then on a higher concentration, when a bias higher than the recommended thresholds was observed on the first concentration. On the Atellica system, icterus is expressed as an index ranging from 0 to 6 (Table 1) (*5*). Lyophilized unconjugated bilirubin (> 98%; Merck, Fontenay sous Bois, France) was dissolved in 0.1 N NaOH, just before each interference testing (*7*-*10*). This sample was vortexed, protected from light by aluminium foil, let stand 5 minutes and vortexed again before the next step. It was added to a fraction of the plasma or serum pool with a dilution factor 1:10 (v/v), and the tube was protected from light until being used for the preparation of spiked samples. Bilirubin concentration was measured on the native pool and on this unconjugated bilirubin-enriched sample on Atellica with dedicated reagent, and, based on these results, we prepared different dilutions in the native pool, to achieve a set of 5 samples with the desired bilirubin concentrations (1 sample per icterus index level). For each level of the icterus index, bilirubin concentration was adjusted to a concentration just below the threshold for the above index level indicated in Table 1 (maximum 10 µmol/L below). At the end, 0.1 N NaOH solution always represented less than 5% (v/v) of the final samples. For each parameter, we checked that 5% (v/v) 0.1 N NaOH did not influence results on native samples (data not shown). For conjugated bilirubin, we purchased bilirubin conjugate ditaurate disodium salt from Merck as described in previous studies (*6*, *11*-*13*). We directly mixed it in a fraction of the plasma or serum pool. This sample was vortexed, let stand 5 minutes and vortexed again before the next step. It was protected from light by aluminium foil until being used for the preparation of spiked samples. Bilirubin concentration was measured on the conjugated bilirubin-enriched pool and then we processed as described for unconjugated bilirubin.

**Table 1:** Correspondence between the icterus index values and bilirubin concentrations on the Atellica system, according to the manufacturer’s specifications (5)

**Index value**	**Bilirubin concentration (µmol/L)**
0	< 34.2
1	34.2-170
2	171-342
3	342-513
4	513-684
5	684-1026
6	> 1027

### Methods

Analyte measurements were performed using an Atellica automated platform equipped with a chemistry module CH930 and an immunoanalysis module IM1300. The methods for the different parameters are detailed in Table 2. Measurements for each analyte were performed in triplicate, as previously done in similar works, within the shortest possible timeframe using the same analyzer and reagent batches (*9*, *14*). Samples were loaded on the analyzer just after their preparation in the following order: native pool, conjugated bilirubin enriched samples from I = 1 to 5, and unconjugated bilirubin enriched samples from I = 1 to 5. Experiments were conducted at a time when activity was low in our department, so analysis were performed very quickly after sample loading. All assays were conducted in accordance with the manufacturer’s instructions, with routine internal and external quality control procedures in place, including for hemolysis, icterus and lipemia indexes (Probioqual, Lyon, France). Our laboratory is accredited according to ISO 15189:2022 standards.

**Table 2:** Methods and wavelengths used for the quantification of the parameters evaluated in this study

	**Methods**		**Wavelengths (nm)**
**CHEMISTRY MODULE**			
Alanine aminotransferase, P5P		Conversion NADH to NAD	340/410
Albumin, bromocresol purple		Complex albumin-bromocresol purple	596/694
Apolipoprotein AI		Immunoturbidimetry	340/694
Apolipoprotein B		Immunoturbidimetry	340/694
Aspartate aminotransferase, P5P		Conversion NADH to NAD	340/410
Calcium		Complex calcium-arsenazo III	658/694
Carbon dioxide		Conversion NADH analog to NAD analog	410/478
Ceruloplasmin		Immunoturbidimetry (Sentinel reagent)	340
Cholesterol, HDL		Trinder, substrate: 4 aminoantipyrine + NNbis-(4-sulfobutyl)-m-toluidine	596/694
Cholesterol, LDL		Trinder, substrate: 4 aminoantipyrine + DSBmT (NNbis-(4-sulfobutyl)-m-toluidine)	545/694
Cholesterol, total		Trinder, substrate: 4 aminoantipyrine + phenol	505/694
Creatinine		Trinder, substrate: 4 aminoantipyrine + N-ethyl-N-(3-methylphenyl)-N’-succinyl-ethylenediamine	545/694
Gamma-glutamyl transferase		Transfer of g-glutamyl group from g-glutamyl-3-carboxy-4-nitroanilide to glycylglycine	410/478
Glucose, hexokinase		Conversion NAD to NADH	340/410
Haptoglobin		Immunoturbidimetry	340/694
Immunoglobulin A		Immunoturbidimetry	340/596
Immunoglobulin G		Immunoturbidimetry	340/694
Lactic acid		Trinder, substrate: 4 aminoantipyrine + proton donor	
Lipase		Product of the substrate transformed by lipase	571/694
Lipoprotein (a)		Immunoturbidimetry	694
Magnesium		Complex with xylitlyl blue	505/694
Phosphorus		Complex phosphomolybdate	340/658
Prealbumin		Immunoturbidimetry	410
Protein, total		Complex with CuSO4	545
Transferrin		Immunoturbidimetry	340/596
Triglyceride		Trinder, substrate: 4 aminoantipyrine + 4 chlorophenol	505/694
Urea		Conversion NADH to NAD	340/410
Uric acid		Trinder, substrate: 4 aminophenazone + N-ethyl-N-[2 hydroxy-3 sulfopropyl]-3 methylaniline]	545/694
**IMMUNOANALYSIS MODULE**			
AFP		Sandwich immunoassay, chemiluminescence, acridinium ester	596
CA 125		Sandwich immunoassay, chemiluminescence, acridinium ester	596
CA 15-3		Sandwich immunoassay, chemiluminescence, acridinium ester	596
CEA		Sandwich immunoassay, chemiluminescence, acridinium ester	596
Cortisol		Competitive immunoassay, chemiluminescence, acridinium ester	596
C-peptide		Sandwich immunoassay, chemiluminescence, acridinium ester	596
Folate		Competitive immunoassay, chemiluminescence, acridinium ester	596
FSH		Sandwich immunoassay, chemiluminescence, acridinium ester	596
fT3		Competitive immunoassay, chemiluminescence, acridinium ester	596
fT4		Competitive immunoassay, chemiluminescence, acridinium ester	596
Insulin		Sandwich immunoassay, chemiluminescence, acridinium ester	596
LH		Sandwich immunoassay, chemiluminescence, acridinium ester	596
Oestradiol		Competitive immunoassay, chemiluminescence, acridinium ester	596
Progesteron		Competitive immunoassay, chemiluminescence, acridinium ester	596
Prolactin		Sandwich immunoassay, chemiluminescence, acridinium ester	596
TSH		Sandwich immunoassay, chemiluminescence, acridinium ester	596
Vitamin B12		Competitive immunoassay, chemiluminescence, acridinium ester	596
The informations are issued from the technical sheets (5). AFP - alpha fetoprotein. CA - carbohydrate antigen. CEA - carcinoembryonic antigen. FSH - follicle stimulating hormone. fT4 - free thyroxin. fT3 - free triiodothyronine. HDL - high density lipoprotein. LDL - low density lipoprotein. LH - luteinizing hormone. P5P - pyridoxal-5-phosphate. TSH - thyroid stimulating hormone.			

### Statistical analysis

The mean of the three replicate values was used for analysis. A correction by the dilution factor was applied for the samples enriched with unconjugated bilirubin. The difference in concentration between each bilirubin-spiked sample and no spiked native pool was expressed in percentage, thereafter referred to as “bias”. In a first approach, we considered that interference was significant when the absolute bias exceeded 10%, in accordance with the process described by Siemens. In addition to this generic threshold, analyte specific acceptability criteria based on biological variation were also applied. Specifically, we evaluated whether the bias exceeded the desirable total allowable analytical error defined by the European Biological Variation Study (EuBIVAS) conducted by the European Federation of Clinical Chemistry and Laboratory Medicine (EFLM) (*15*, *16*).

## Results

### Estimation of bilirubin-induced interference

All the results are summarized in Table 3 and the results showing an interference higher than EuBIVAS threshold are graphically represented on Supplementary figures 1 and 2. For 29 of the 45 parameters tested, no bias above 10% was observed up to I = 5, for conjugated or unconjugated bilirubin. For 26 of these 29 parameters, the bias was also lower than EuBIVAS threshold (no recommendation for C-peptide). For the 16 remaining parameters a bias higher than 10% was observed for I-index ≤ 5, either for conjugated bilirubin, unconjugated bilirubin or both. For all of these parameters except for progesteron, similar results were observed with EuBIVAS thresholds. For aspartate aminotransferase, low density lipoprotein (LDL) cholesterol, uric acid, creatinine, total protein, folate, and estradiol, the bias was higher with conjugated bilirubin compared to unconjugated bilirubin. On the contrary, for calcium, total cholesterol, triglycerides, free triiodothyronine (fT3), free thyroxine (fT4), follicle - stimulating hormone (FSH), progesteron and vitamin B12, the bias was higher with unconjugated bilirubin. For lactate, both forms of bilirubin had similar effects. On the other hand, for the different parameters cited above, except for creatinine and uric acid, the bias decreased when the concentration of the analyte increased, falling well below 10% in some cases.

**Table 3:** Biases induced by interference of conjugated and unconjugated bilirubin

**Analyte (matrix)**	**Baseline concentration**	**Bias relative to baseline following spiking (%)** **Conjugated bilirubin** **Unconjugated bilirubin**					**EuBIVAS acceptable bias (desirable performance) (%) (14, 15)**
		I = 1	I = 2	I = 3	I = 4	I = 5	
**CHEMISTRY MODULE**							
Alanine aminotransferase, P5P (Ph)	49.3 IU/l	+ 4.8 - 3.8	+ 4.8 - 5.7	+ 4.1 - 7.8	+ 1.4 - 7.6	- 0.6 + 3.9	18.7
Albumin, bromocresol purple (Ph)	25.7 g/L	+ 1.2 - 0.4	+ 1.2 + 0.4	- 2.7 + 3.8	- 2.7 + 2.0	+ 1.2 + 2.4	3.3
Apolipoprotein AI (S)	1.20 g/L	NP NP	NP NP	- 1.1 + 0.4	- 1.1 - 0.3	- 0.8 + 1.1	6.2
Apolipoprotein B (S)	0.74 g/L	NP NP	NP NP	+ 0.5 - 0.5	- 0.5 0.0	0.0 + 1.6	10.6
Aspartate aminotransferase, P5P (Ph)	46.3 IU/L	+ 10.2 + 1.1	+ 18.1 + 3.1	+ 30.3 + 1.4	+ 38.2 - 0.4	+ 48.3 + 2.4	12.4
	187 IU/L	+ 3.7 + 0.5	+ 6.0 0.0	+ 8.3 - 1.0	+ 12.8 - 1.4	+ 16.4 + 0.4	
Calcium (Ph)	1.93 mmol/L	+ 1.9 - 0.1	+ 1.1 - 4.6	- 0.7 - 6.1	+ 0.2 - 7.2	+ 0.4 - 13.1	2.3
	2.63 mmol/L	- 0.3 - 2.0	+ 0.9 - 2.3	- 0.4 - 2.5	+ 0.1 - 3.9	- 0.9 - 4.2	
Carbon dioxide (Ph)	16 mmol/L	0.0 - 1.1	- 2.1 - 0.1	- 2.1 - 2.3	- 2.1 - 3.0	- 6.3 - 4.2	4.9
	28 mmol/L	- 1.2 + 1.1	- 1.2 + 3.4	0.0 + 2.7	0.0 + 3.8	0.0 - 0.4	
Ceruloplasmin (S)	0.20 g/L	- 1.7 - 0.7	0.0 + 0.3	- 1.7 + 3.1	- 1.7 + 4.2	- 5.0 + 5.0	8.1
Cholesterol, HDL (S)	0.76 mmol/L	- 0.4 + 3.7	+ 3.1 + 5.6	+ 2.2 + 6.7	- 4.8 + 6.5	- 5.3 + 6.2	9.9
Cholesterol, LDL (S)	1.68 mmol/L	- 3.6 + 0.2	- 8.9 - 1.6	- 15.1 - 6.5	- 19.8 - 8.2	- 30.0 -11.2	11.8
	3.19 mmol/L	- 2.1 - 0.7	- 5.0 - 0.9	- 7.4 - 4.3	- 9.9 - 5.3	- 17.2 - 7.5	
Cholesterol, total (S)	2.87 mmol/L	+ 3.7 + 13.1	+ 5.3 + 23.3	+ 5.6 + 34.4	+ 6.3 + 40.8	+ 7.2 + 48.9	8.3
	4.90 mmol/L	+ 0.5 + 3.0	- 1.0 + 7.5	- 1.5 + 13.0	- 1.6 + 14.1	- 2.7 + 17.9	
Creatinine (Ph)	67.7 µmol/L	- 4.0 + 1.5	- 7.4 - 0.7	- 7.9 - 5.6	- 14.8 - 4.2	- 18.3 - 9.8	7.8
	213 µmol/L	- 2.2 + 0.2	- 6.1 - 0.6	- 8.9 - 2.1	- 13.3 - 4.1	- 15.8 - 4.1	
Gamma-glutamyl transferase (Ph)	IU/L	- 0.7 - 2.2	- 0.7 - 4.8	0.0 - 3.9	- 2.2 - 6.6	- 5.1 - 6.4	18.3
Glucose (Ph)	4.59 mmol/L	- 0.1 + 0.4	- 1.3 + 0.5	- 1.5 - 0.5	- 2.2 + 1.5	- 2.5 + 0.4	6.1
Haptoglobin (Ph)	0.30 g/L	- 1.1 0.0	- 2.2 - 1.0	- 2.2 - 2.7	- 2.2 - 2.0	- 3.3 0.0	17.1
Immunoglobulin A (S)	0.72 g/L	- 0.5 - 0.4	- 0.9 - 1.7	- 3.2 - 2.1	- 4.9 - 1.1	- 2.8 + 1.5	14.5
Immunoglobulin G (S)	6.70 g/L	- 0.0 + 0.3	+ 3.4 + 1.1	+ 3.3 + 4.4	+ 0.3 + 5.1	+ 0.1 + 2.3	7.3
Lactic acid (Pf)	1.88 mmol/L	- 11.0 - 9.7	- 12.8 - 11.0	- 51.1 - 33.5	- 51.8 - 41.8	- 53.2 - 59.7	36.2
	8.51 mmol/L	- 1.3 - 0.6	- 2.2 - 2.2	- 3.4 - 4.7	- 4.6 - 4.6	-7.6 - 8.9	
Lipase (Ph)	48.7 IU/L	- 0.3 + 0.7	- 1.7 + 0.3	- 3.1 - 1.3	- 0.3 + 0.6	- 2.1 - 4.5	12.9
Lipoprotein (a) (S)	157 mg/L	+ 1.0 + 1.3	+ 0.8 + 2.8	+ 1.5 + 1.2	+ 0.8 + 1.8	+ 0.6 + 4.5	29.8
Magnesium (Ph)	0.75 mmol/L	- 1.3 - 0.3	- 1.8 - 1.1	- 3.6 - 3.4	- 4.9 - 4.7	- 6.2 - 6.0	3.8
	1.16 mmol/L	- 0.6 + 0.8	- 0.9 - 0.2	- 1.7 - 2.9	- 2.9 - 2.7	- 4.9 - 1.1	
Phosphorus (Ph)	1.08 mmol/L	0.0 + 0.7	+ 1.9 - 0.8	+ 2.5 - 0.9	+ 4.0 - 0.1	+ 7.4 - 1.9	9.6
Prealbumin (Ph)	0.19 g/L	+ 1.8 + 2.0	1.8 1.0	- 3.5 + 0.5	- 3.5 - 2.1	- 5.3 + 0.8	14.5
Total protein (Ph)	49.6 g/L	- 4.8 - 4.5	- 10 - 8.4	- 17.3 - 12.2	- 22.3 - 16.0	- 34.2 - 22.0	3.8
	72.8 g/L	- 3.3 - 2.0	- 5.3 - 4.1	- 9.0 - 6.0	- 11.3 - 7.9	- 17.0 - 11.6	
Transferrin (S)	1.86 g/L	+ 0.5 + 0.8	+ 0.2 + 2.6	+ 0.9 + 2.5	- 0.2 + 1.5	+ 1.6 + 1.6	6.8
Triglycerides (S)	1.12 mmol/l	+ 2.1 + 5.5	+ 3.6 + 17.8	+ 3.3 + 34.9	+ 5.1 + 40.1	+ 6.5 + 53.8	25.8
	2.80 mmol/L	- 0.1 + 7.0	2.6 + 11.8	4.3 + 18.3	- 6.5 + 20.4	- 9.6 + 25.6	
Urea (Ph)	6.3 mmol/L	+ 1.6 + 1.0	+ 2.6 + 1.2	+ 2.1 + 0.9	+ 1.1 - 3.1	+ 4.2 - 1.5	17.1
Uric acid (Ph)	310 mmol/l	- 6.8 - 0.3	- 14.2 - 0.8	- 24.6 - 0.4	- 32.5 - 0.5	- 55.3 - 1.0	11.6
	508 mmol/L	- 6.3 - 1.0	- 14.6 - 0.9	- 25.7 - 0.7	- 31.1 - 1.0	- 45.5 - 0.6	
**IMMUNOANALYSIS MODULE**							
AFP (S)	10.9 µg/L	NP NP	NP NP	+ 3.4 + 1.2	+ 2.8 + 8.0	+ 0.9 - 2.8	17,6
CA125 (S)	37.2 kIU/L	NP NP	NP NP	+ 7.5 - 1.5	+7.8 - 1.1	+ 8.6 - 3.1	13,9
CA15-3 (S)	37.3 kIU/L	NP NP	NP NP	+ 3.6 - 1.3	+ 5.5 - 0.8	+ 7.0 + 0.7	12,9
CEA (S)	4.4 µg/L	NP NP	NP NP	0.0 - 4.7	+ 6.1 + 2.1	+ 2.3 - 2.4	26,9
Cortisol (S)	5.69 µg/dL	NP NP	NP NP	NP NP	+ 4.2 + 4.7	+ 9.8 + 4.7	22.2
C-peptide (S)	0.41 ng/mL	NP NP	NP NP	+ 3.3 - 0.9	+ 2.4 + 1.0	+ 4.1 + 10.1	none
Folate (S)	3.14 ng/mL	+ 8.0 NP	+ 10.0 NP	+ 15.6 - 5.1	+ 18.2 - 3.6	+ 16.7 - 4.8	15.5
	5.48 ng/mL	+1.0 NP	+ 5.8 NP	+ 9.4 - 4.5	+ 13.1 - 3.2	+ 10.1 - 2.4	
FSH (S)	4.8 IU/L	0.0 + 3.8	- 1.4 - 2.5	+ 3.5 + 23.1	+ 2.8 + 22.9	- 2.8 + 20.6	17.6
	71 IU/L	NP NP	NP NP	+ 8.5 + 4.3	+ 9.4 + 3.5	+ 2.8 + 6.7	
fT4 (Ph)	9.4 pmol/L	+ 0.7 + 4.6	- 1.1 + 7.2	- 1.1 + 12.2	0.0 + 17.3	+ 3.2 + 27.1	6.3
	15.0 pmol/L	+ 0.9 + 2.6	+ 0.4 + 3.8	+ 1.1 + 9.9	+ 6.2 + 7.2	+ 2.7 + 12.7	
fT3 (Ph)	2.83 pmol/L	- 1.1 + 3.4	+ 1.3 + 6.9	+ 0.1 + 9.0	- 3.4 + 10.9	- 6.9 + 16.5	11.3
	5.7 pmol/L	- 1.8 - 0.2	- 4.1 + 1.1	- 5.3 + 5.4	- 9.4 + 5.3	- 10.5 + 7.7	
Insulin (S)	3.00 mIU/L	NP NP	NP NP	NP NP	NP NP	+ 1.1 + 2.4	31.5
LH (S)	2.9 IU/L	NP NP	NP NP	0.0 + 3.0	- 2.3 + 2.3	- 6.9 - 0.8	29.1
Oestradiol (S)	135 ng/L	+ 3.7 - 1.7	+ 5.7 - 5.3	+ 12.6 - 9.0	+ 23.2 - 8.0	+ 25.4 - 8.9	18.3
	1294 ng/L	+ 1.4 + 2.8	- 1.2 + 3.9	- 5.8 + 3.6	- 4.8 + 6.1	- 7.3 + 5.1	
Progesteron (S)	1.16 µg/L	+ 0.6 + 2.2	- 1.7 - 0.9	- 3.4 - 0.4	- 4.3 - 2.7	- 12.1 - 11.4	26.2
	9.74 µg/L	- 1.4 - 1.3	- 1.0 - 4.6	- 3.8 - 7.8	- 5.5 - 19.1	- 8.0 - 22.3	
Prolactin (S)	246 mIU/L	NP NP	NP NP	- 5.1 - 7.7	- 4.2 - 8.2	- 5.3 - 5.4	56.7
TSH (Ph)	2.08 mIU/L	NP NP	NP NP	+ 0.3 - 3.3	+ 0.6 - 2.0	+ 1.0 + 1.0	24.8
Vitamin B12 (S)	244 ng/L	NP NP	NP NP	+ 3.3 + 2.7	+ 3.6 + 5.1	+ 3.6 + 13.3	15.4
	1376 ng/L	NP NP	NP NP	+ 0.7 - 1.5	+1.0 + 1.0	+ 2.3 + 4.3	
Results are from measurements performed in triplicate and averaged. Biases are calculated as the difference between spiked and native samples and expressed in %. AFP - alpha fetoprotein. CA carbohydrate antigen. CEA - carcinoembryonic antigen. FSH - follicle stimulating hormone. fT4 - free thyroxin. fT3 - free triiodothyronine. HDL - high density lipoprotein. I - icterus index. LDL - low density lipoprotein. LH - luteinizing hormone. NP - not performed. P5P - pyridoxal-5-phosphate. P_h_ - heparinized plasma. P_f_ - sodium fluoride plasma. S - serum. TSH - thyroid stimulating hormone.							

### Revised I-index thresholds

Table 4 lists the manufacturer-suggested I-index thresholds. An icterus-induced bias was proven to be higher than 10% only for a few of the parameters. For most of parameters, the bias was below 10%, but tests were not performed for higher bilirubin concentrations. In this case, as a precaution, the manufacturer considered that the bias could be more than 10% above the tested threshold. We suggest new thresholds for I-index, based on our experimental results, firstly considering bias above 10% as significant. For 29 parameters, I-index threshold can be increased to 6 instead of 2 to 5 (Table 4). For creatinine, fT3 and progesteron, it can be increased from 3 to 4, and for vitamin B12 it can be set at 5 instead of 3. At the opposite, based on a significant interference for bias higher than 10%, the threshold for aspartate aminotransferase, total cholesterol, and lactate should be stricter than those suggested by the manufacturer. Based on EuBIVAS desirable recommended performance limits reported in Table 3, the index threshold can be maintained at 6 for parameters with an icterus-induced interference below 10%, except for carbon dioxide (I = 5) and magnesium (I = 4). For parameters with a bias exceeding 10%, we also suggested I-index thresholds related to EuBIVAS recommended performance limits. These indexes were less strict than those based on a bias higher than 10% for 7 parameters, similar for 5 and stricter for 4.

**Table 4:** Suggested revised index thresholds for icterus interference

	**Index suggested by Siemens**	**Revised index for bias ≤ 10%**	**Revised index for bias ≤ EuBIVAS thresholds**
**CHEMISTRY MODULE**			
Alanine aminotransferase, P5P	3*	6	6
Albumin, bromocresol purple	4*	6	6
Apolipoprotein AI	4*	6	6
Apolipoprotein B	4*	6	6
Aspartate aminotransferase, P5P	3*	1	2
Calcium	5*	5	2
Carbon dioxide	4*	6	5
Ceruloplasmin	4*	6	6
Cholesterol, HDL	4*	6	6
Cholesterol, LDL	3	3	3
Cholesterol, total	3	1	1
Creatinine	3*	4	3
Gamma-glutamyl transferase	2*	6	6
Glucose	4*	6	6
Haptoglobin	3*	6	6
Immunoglobulin A	3*	6	6
Immunoglobulin G	5*	6	6
Lactic acid	2	1	3
Lipase	3*	6	6
Lipoprotein (a)	3*	6	6
Magnesium	4*	6	4
Phosphorus	4*	6	6
Prealbumin	3*	6	6
Total protein	3*	3	1
Transferrin	4*	6	6
Triglycerides	2*	2	3
Urea	3*	6	6
Uric acid	2*	2	2
**IMMUNOANALYSIS MODULE**			
AFP	3*^†^	6	6
CA125	3*^†^	6	6
CA15-3	3*^†^	6	6
CEA	3*^†^	6	6
Cortisol	3*^†^	6	6
C-peptide	3*^†^	6	none
Folate	3	3	3
FSH	3*^†^	3	3
fT4	3*^†^	3	2
fT3	3*	4	5
Insulin	3*^†^	6	6
LH	3*	6	6
Oestradiol	3*^†^	3	4
Progesteron	3*	4	6
Prolactin	3*^†^	6	6
TSH	5*	6	6
Vitamin B12	3*^†^	5	6
*indicates that the manufacturer did not show significant interference for the index below but that interference assays for that index and higher have not been reported in the technical sheets. ^†^indicates that information has been extrapolated from the Centaur analyzer (Siemens), without any experiment performed on Atellica. AFP - alpha fetoprotein. CA - carbohydrate antigen. CEA - carcinoembryonic antigen. FSH - follicle stimulating hormone. fT4 - free thyroxin. fT3 - free triiodothyronine. HDL - high density lipoprotein. LDL - low density lipoprotein. LH - luteinizing hormone. P5P - pyridoxal-5-phosphate. TSH - thyroid stimulating hormone.			

## Discussion

The present study performed on the Atellica platform (Siemens) highlights the need for revised thresholds for significant icterus-induced interference in most of the parameters studied. Among the 45 tested parameters, a new threshold was suggested for 36 of them using the 10% limit method, and for 38 using the EuBIVAS limit method. In addition, our findings highlight the differential impact of conjugated and unconjugated bilirubin, and possibly the dependence of icterus-induced interference on the concentration of the measured parameter on Atellica analyzers. To the best of our knowledge, no similar work has been previously reported for the Atellica platform, in contrast to published studies on the Alinity (Abbott) and Cobas (Roche) analyzers (*13*, *17*).

Acceptability limits have been defined by different ways throughout the literature (*6*, *8*, *14*). The Atellica manufacturer chose 10% for all parameters, and we initially kept the same criteria in line with this choice, so that our work could be seen as the extension of manufacturer’s work. However, as suggested by Von Meyer *et al.*, a single 10% threshold does not take into account within-subject and within-group biological variability, and is not always the most appropriate approach in a context of medical decision (*4*). Several acceptability limits are often proposed for one parameter, as can be seen in the synthetic document on the Westgard Quality Control site, which may make the certain choice difficult (*15*). We also proposed revised I-index thresholds based on the allowable total errors issued from EuBIVAS conducted by the European Federation of Clinical Chemistry and Laboratory Medicine. Although the revised I-index threshold is similar for both evaluations for many parameters, it differs for others. For biochemical parameters such as magnesium and calcium which have a narrow biological variation and a weak icterus interference, the proposed I-index is lower with EuBIVAS threshold compared to the 10% threshold. On the contrary, for hormonal parameters impacted by icterus, I-index threshold is likely to be higher based on EuBIVAS criteria due to considerable biological variations.

In most cases, the threshold for significant icterus-induced interference was increased, while it was reduced for a few analytes. Knowing the precise impact of interferences on laboratory results is essential for interpretation and patient care. The major issue for Atellica users is that the manufacturer did not evaluate icterus interference across the full range of the bilirubin concentrations likely to be encountered in patients and suggests, as a precautionary measure, to consider that a significant interference may occur above the tested threshold. In addition, the range of bilirubin concentration tested is highly variable from one parameter to another, stopping sometimes at very low concentrations. Very interestingly, our work demonstrated that for many parameters reliable results can be released for all patients, since no bias higher than 10% or EuBIVAS recommended threshold was observed for bilirubin concentration reaching 1000 µmol/L, which is almost never encountered in clinical practice. Not releasing these results from I-index equal to 3 or 4 (equivalent to a bilirubin concentration equal to 340 or 510 µmol/L, respectively), or releasing them with a doubt about a potential interference is an issue especially in hospital where patients with an icterus are frequent. In addition, we suggested revised I-index thresholds lower than 6 but with a value higher than that suggested by the manufacturer for some parameters. Replacing the manufacturer’s thresholds by the thresholds based on our results allows to release much more results with confidence without any doubt about a potential bilirubin-induced interference. In case of a significant icterus interference, laboratory professionals choose between cancelling the result or releasing it with a comment. Our work provides further insight for formulating a potential comment with the sense of interference and an estimation of it.

We performed assays with both conjugated and unconjugated bilirubin which showed that interference may be very different for the two forms of bilirubin. This has previously been reported for different parameters, underlying the importance of performing tests with both forms of bilirubin (*11*, *18*). In practice, the measurement of icterus indices does not allow to tell the difference between them, and in an automated work process, some results are wrongly tagged or even cancelled. For these parameters, a further analysis of the results including the measurement of each of the two forms of bilirubin could lead to the release of reliable results not biased by any interference, in case of the increase in the bilirubin fraction without any interference. Thus, our data are also important for guiding the transmission of results in patients with a broad predominance of one form of bilirubin. The reasons for the differential interference of conjugated and unconjugated bilirubin are not clear. Interestingly, Table 2 shows that, among the methods with the Trinder reaction, those using phenol or a derivative as a chromogen with a reading at 505/694 nm, present a positive interference mainly with unconjugated bilirubin. On the contrary, those using another chromogen with reading at 545/694 nm present a negative interference mainly with conjugated bilirubin. Negative interference of bilirubin is frequently mentioned in line with its reducing power. The sense of interference and the form of interfering bilirubin appears to depend on the nature of chromogen in the Trinder reaction. Thus, for a single parameter, interference may be variable and cannot always be anticipated, which is the case for triglycerides for example (*6*, *7*). As far as immunoassays are concerned, icterus-induced interference occurs mainly with competitive assays. Again, the mechanism of interference is not clear since it is alternatively observed with conjugated or unconjugated bilirubin. Exposure of an acridinium ester label to an alkaline hydrogen peroxide solution triggers a flash of light, and bilirubin may reduce hydrogen peroxide. The bias is usually positive. For competitive immunoassays, this may be consistent with the reducing power of bilirubin which interferes with the oxidoreduction reaction of acridinium ester. Another explanation may be that bilirubin disrupts the binding of the competitor to the antibody.

The main limitation of this study concerns parameters for which icterus-induced interference is very different for conjugated and unconjugated bilirubin. Indeed, analyzers do not differentiate both forms of bilirubin and the recommended I-index thresholds, based on the bilirubin form with the highest interference, may lead to flag results in an abusive manner. However, in patients with a broad predominance of one form, results may be reinterpreted by the laboratory professionals.

In conclusion, this study provides many relevant additional data concerning bilirubin-induced interference on the quantification of 45 parameters on Atellica analyzers CH930 and IM1300. Our findings can be used to improve the interpretation of results in patients with increased blood bilirubin concentration and are of major importance for the daily practice of clinical laboratories equipped with Atellica analyzers. Indeed, much more results can be released with confidence without any doubt about a potential bilirubin-induced interference. In addition, knowing the differential impact of both forms of bilirubin on some parameters gives the opportunity to interpret more specifically the impact on results for the patients with a hyperbilirubinemia with a broad predominance of one form of bilirubin. Also, knowing that bilirubin-induced interference is modulated by the concentration of the analyte for some parameters may differentially guide the release of results.

## Data Availability

The data generated and analyzed in the presented study are available from the corresponding author on request.
